# Inhibitory Effects of 3-Deoxysappanchalcone on Particulate-Matter-Induced Pulmonary Injury

**DOI:** 10.3390/cimb47080608

**Published:** 2025-08-01

**Authors:** Chang-Woo Ryu, Jinhee Lee, Gyuri Han, Jin-Young Lee, Jong-Sup Bae

**Affiliations:** 1 College of Pharmacy, CMRI, Research Institute of Pharmaceutical Sciences, Kyungpook National University, Daegu 41566, Republic of Korea; ryu76kr@daum.net (C.-W.R.); aadd8563@gmail.com (J.L.); f11074@naver.com (G.H.); 2 Department of Biological Sciences, College of Natural Sciences, Keimyung University, Daegu 42601, Republic of Korea

**Keywords:** 3-deoxysappanchalcone, particulate matter, vascular permeability, Akt

## Abstract

Fine particulate matter (PM_2.5_) exposure has been linked to increased lung damage due to compromised vascular barrier function, while 3-deoxysappanchalcone (3-DSC), a chalcone derived from *Caesalpinia sappan*, is known for its pharmacological benefits such as anti-cancer, anti-inflammatory, and antioxidant effects; however, its potential role in mitigating PM_2.5_-induced pulmonary damage remains unexplored. To confirm the inhibitory effects of 3-DSC on PM_2.5_-induced pulmonary injury, this research focused on evaluating how 3-DSC influences PM_2.5_-induced disruption of the barrier of the endothelial cells (ECs) in the lungs and the resulting pulmonary inflammation. Permeability, leukocyte migration, proinflammatory protein activation, reactive oxygen species (ROS) generation, and histology were assessed in PM_2.5_-treated ECs and mice. This study demonstrated that 3-DSC effectively neutralized the reactive oxygen species (ROS) generated by PM_2.5_ exposure in the lung endothelial cells, suppressing ROS-triggered p38 MAPK activation while enhancing Akt signaling pathways critical to preserving vascular barrier function. In animal models, 3-DSC administration markedly decreased vascular permeability, attenuated the influx of immune cells into the lung tissue, and lowered inflammatory mediators like cytokines in the airways of PM_2.5_-exposed mice. These data suggest that 3-DSC might exert protective effects on PM_2.5_-induced inflammatory lung injury and vascular hyperpermeability.

## 1. Introduction

Epidemiological research highlights particulate matter (PM) as a critical global health concern, with studies linking chronic PM exposure to a reduced life expectancy and organ-specific damage, particularly in the lungs. Airborne PM_2.5_ particles under 2.5 μm pose unique risks as a pervasive pollutant, correlating with cardiovascular conditions like arteriosclerosis, chronic respiratory illnesses, and an increased incidence of lung cancer [1,2]. Its ultrafine particles evade respiratory defenses, infiltrating the alveolar regions, while surface-bound contaminants such as heavy metals, polycyclic aromatic hydrocarbons, and endotoxins exacerbate toxicity through oxidative stress and inflammatory pathways [3,4]. Given the slow progress in environmental remediation, researchers emphasize the urgent need to develop innovative interventions protecting against PM-induced respiratory damage.

Plant-based medicine presents a compelling alternative for anticoagulant therapy, leveraging bioactive compounds with fewer adverse effects [5]. Among the phenolic compounds extracted from the heartwood of *Caesalpinia sappan* L., 3-deoxysappanchalcone (3-DSC) stands out, alongside other bioactive molecules such as brazilin, protosappanin, and homoisoflavonoids, all known for their diverse pharmacological properties [6]. Extracts from *C. sappan* have been shown to possess therapeutic benefits, including anti-inflammatory, antioxidant, anti-allergic, anti-influenza, hepatoprotective, and immunomodulatory effects [7,8,9,10,11], with toxicological studies confirming their safety [12]. Given that 3-DSC is a key compound in *C. sappan* and exhibits anti-inflammatory and immunomodulatory activities, we proposed that it could mitigate PM_2.5_-induced lung damage. To test this hypothesis, we employed a mouse model to assess the impact of 3-DSC on lung histopathology, inflammation, and oxidative stress following PM_2.5_ exposure and investigated its ability to counteract PM_2.5_-induced vascular barrier dysfunction in an in vitro system.

## 2. Materials and Methods

### 2.1. Reagents

3-DSC, with a purity exceeding 95%, was sourced from MuseChem (Fairfield, NJ, USA), while Diesel PM NIST 1650b (PM_2.5_) was procured from Sigma-Aldrich (St. Louis, MO, USA), suspended in saline, and subjected to 30 min of sonication to prevent particle aggregation. High-molecular-weight hyaluronan from Sigma-Aldrich served as the positive control [13]. Unless specified otherwise, all additional reagents and chemicals were supplied by Sigma-Aldrich. The authors confirm that the principal investigator for this paper was Jong-Sup Bae and that he took direct clinical responsibility for the research subjects.

### 2.2. Animals and Husbandry

Seven-week-old male Balb/c mice, weighing around 27 g, were obtained from Orient Bio Co. (Sungnam, Republic of Korea) and acclimated for 12 days before experimentation. The mice were housed in groups of five per polycarbonate cage under controlled environmental conditions, including a temperature range of 20–25 °C, humidity levels of 40–45%, and a 12 h light/dark cycle. All of the procedures followed the animal care guidelines established by Kyungpook National University (IRB No. KNU 2017-101). The mice received oral doses of 3-DSC (0.05–0.4 mg/kg) or hyaluronan (0.1%) daily for 10 days before being intratracheally exposed to PM_2.5_ (1 mg/kg in 100 µL of saline), as described by Wang et al. [14]. After 10 days of PM_2.5_ exposure, the mice were euthanized, and bronchoalveolar lavage fluid (BAL) along with lung tissues were collected for subsequent analysis.

### 2.3. Primary Culture of the Mouse Lung Microvascular Endothelial Cells

Mouse lung microvascular endothelial cells (MLMVECs) were isolated following a previously established method [15]. The lung tissues were finely chopped and enzymatically digested with collagenase A (1 mg/mL) for 45–60 min at 37 °C. The endothelial cells were then separated using magnetic beads coated with an anti-PECAM-1 monoclonal antibody (BD Pharmingen, San Diego, CA, USA) and cultured for two days in a growth medium. For monolayer cultures, the cells were seeded onto fibronectin-coated plates in an endothelial cell basal medium supplemented with EGM-2 MV BulletKit™ (Lonza, Walkersville, MD, USA) and maintained at 37 °C in a humidified incubator with 5% CO_2_ and 95% air.

### 2.4. The Permeability Assay

To evaluate endothelial cell permeability in vitro, a modified two-compartment chamber system was used to measure the movement of Evans-blue-bound albumin across confluent cell monolayers exposed to varying concentrations of the test compounds, as described previously [16]. Mouse lung microvascular endothelial cells (MLMVECs) were seeded at a density of 5 × 10^4^ cells per well into transwell inserts with a pore size of 3 µm and a diameter of 12 mm; cultured for three days; and treated with 3-DSC for six hours before exposure to PM_2.5_ (1 mg/mL) for another six hours. Permeability measurements were made using an ELISA plate reader based on established protocols [16].

For the in vivo permeability analysis, mice were exposed to PM_2.5_ and treated with the test compounds for ten days. During the procedure, the mice breathed spontaneously and were anesthetized using 2% isoflurane delivered via a rodent gas anesthesia machine (RC2; Vetequip, Pleasanton, CA, USA) and a facemask. A solution of 1% Evans blue dye in saline was injected intravenously, and after six hours, the mice were euthanized through cervical dislocation. Peritoneal fluid samples were collected to assess vascular permeability using previously validated methods [17].

### 2.5. The Leukocyte Migration Assay

Leukocyte infiltration was analyzed by euthanizing the mice after 6 h and flushing the bronchoalveolar space with 5 mL of saline. A 20 µL sample of the lavage fluid was combined with 0.38 mL of Turk’s solution (0.01% crystal violet in 3% acetic acid), and leukocytes were counted using a light microscope.

### 2.6. ELISA for the Assay of Phosphorylated p38 Mitogen-Activated Protein Kinase, Tumor Necrosis Factor-κ, and Interleukin-6

Phosphorylated p38 MAPK levels were analyzed via a commercial ELISA assay (Cell Signaling Technology, Danvers, MA, USA) following the manufacturer’s protocol, while the interleukin-6 (IL-6) and tumor necrosis factor-alpha (TNF-α) concentrations in the bronchoalveolar lavage fluid were quantified using ELISA kits from R&D Systems (Minneapolis, MN, USA). All of the absorbance readings were acquired using a Tecan microplate reader (Tecan Group Ltd., Salzburg, Austria).

### 2.7. The Cell Viability Assay

The cell viability was evaluated using the MTT assay (3-(4,5-dimethylthiazol-2-yl)-2,5-diphenyltetrazolium bromide) to assess the metabolic activity in purified mouse lung microvascular endothelial cells (MLMVECs) isolated from the 3-DSC-treated mice [18,19]. The cells were seeded into 96-well plates at 5 × 10^3^ cells/well and incubated for 24 h. After washing, 100 μL of MTT reagent (1 mg/mL) was added to each well and incubated for 4 h. Formazan crystals formed during the reaction were dissolved with 150 μL of dimethyl sulfoxide (DMSO), and the absorbance at 540 nm was measured using a Tecan microplate reader (Tecan Group Ltd., Austria) to quantify the cell viability.

### 2.8. The Detection of Intracellular ROS

The reactive oxygen species (ROS) levels in the MLMVECs were measured using fluorescence microscopy, following the protocol outlined by Piao et al. [20]. Primary MLMVECs were cultured on 4-well glass chamber slides until they reached over 90% confluence and then incubated with 10 μM of the fluorogenic dye 2′,7′-dichlorofluorescein diacetate (DCFDA; Molecular Probes, Eugene, OR, USA) for 30 min. After removing the DCFDA-containing medium and rinsing the cells, fluorescence microscopy was used to capture images of the stained cells.

### 2.9. The Western Blot Analysis

To perform the Western blot analysis, the cells were washed with chilled phosphate-buffered saline (PBS) and lysed using a buffer containing 0.5% sodium dodecyl sulfate, 1% NP-40, 1% sodium deoxycholate, 150 mM NaCl, 50 mM Tris-HCl (pH 7.5), and protease inhibitors. The protein levels of phosphorylated and total Akt MAPK were detected using specific antibodies from Cell Signaling Technology (Danvers, MA, USA) following the standard Western blot procedures.

### 2.10. Hematoxylin and Eosin Staining

To assess morphological alterations in the lungs, tissues were excised, rinsed thrice with PBS (pH 7.4) to eliminate residual blood, and fixed in 4% formaldehyde (Junsei, Kyoto, Japan) at 4 °C for 20 h. Following fixation, the tissues underwent dehydration using a graded ethanol series before paraffin embedding and microtome sectioning into 4 µm slices. Mounted slides were deparaffinized at 60 °C, rehydrated, and stained with Mayer’s hematoxylin (Sigma-Aldrich). Residual staining was eliminated via brief immersion in 0.3% acidic alcohol, followed by eosin counterstaining (Sigma-Aldrich). The slides were cleared using an ascending ethanol gradient and xylene before coverslipping. A blinded pathologist evaluated the lung histoarchitecture, edema severity, and leukocyte infiltration using light microscopy following the established criteria [21].

### 2.11. The Wet/Dry Weight Ratio of the Lung Tissue

The wet weight of the right lung was recorded, followed by drying the lung for 24 h at 120 °C in an oven to obtain its dry weight. Subsequently, the lung tissue’s W/D weight ratio was calculated to assess lung edema.

### 2.12. The Statistical Analysis

All of the experiments were conducted independently at least three times, with the results presented as the mean values ± standard deviation (SD). Prior to the statistical analysis, data normality was assessed using the Shapiro–Wilk test. As all datasets were confirmed to be normally distributed, statistical differences were analyzed using Student’s *t*-test, with the significance set at *p*-values less than 0.05. The data analysis was performed using SPSS software version 16.0 (SPSS Inc., Chicago, IL, USA).

## 3. Results

### 3.1. The Effects of 3-DSC on PM_2.5_-Mediated Vascular Barrier Disruption

**Figure 1:**
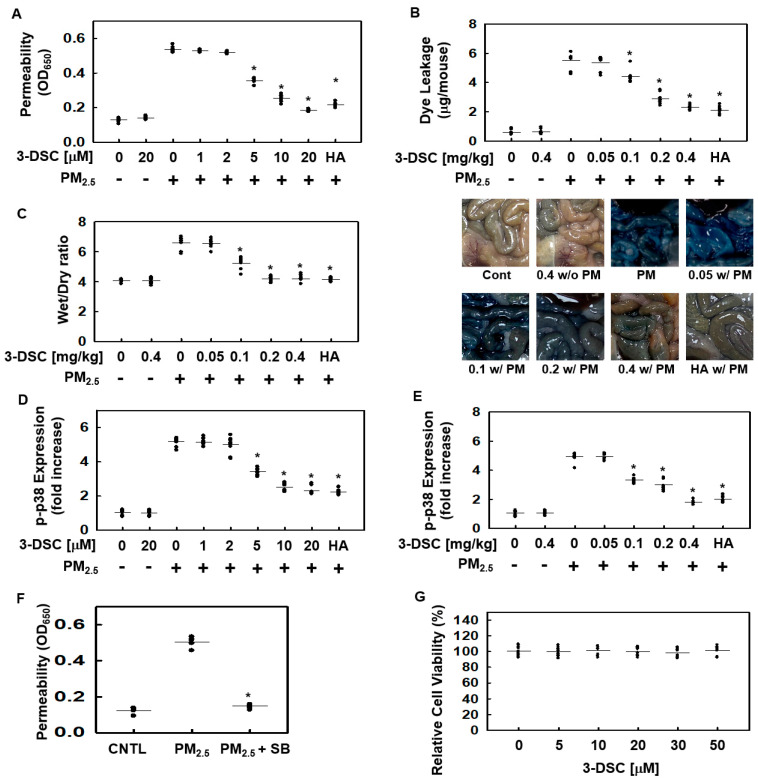
The effects of 3-DSC on particulate matter (PM)-induced endothelial cell (EC) barrier disruptive responses and p38 MAPK activation. (**A**) Mouse lung microvascular endothelial cells (MLMVECs) were pretreated with 3-deoxysappanchalcone (3-DSC; 5, 10, 20, or 50 μM) or hyaluronan (HA; 0.1%) for 6 h, followed by exposure to PM_2.5_ (1 mg/mL) for an additional 6 h. Endothelial barrier dysfunction was evaluated by measuring the permeability for Evans-blue-bound albumin. (**B**,**C**) Male Balb/c mice (7 weeks old, n = 5 per group) were orally administered 3-DSC (0.05, 0.1, 0.2, or 0.4 mg/kg) or HA (0.1%) in vivo daily for 10 days before intratracheal instillation of PM_2.5_ (1 mg/kg in 100 μL saline) for 10 days. Vascular permeability was assessed by quantifying the leakage of Evans blue dye (**B**), and the effects of 3-DSC on the wet-to-dry (W/D) ratio were evaluated (**C**). After intravenous injection of the Evans blue dye, the solution that leaked from the lungs was collected and photographed. The images demonstrated the extent of vascular permeability in response to the experimental treatments by visualizing the intensity of the dye present in the solution obtained from the lungs (**B**). (**D**,**E**) Phosphorylated p38 MAPK levels were measured in MLMVECs (**D**) and in purified MLMVECs from treated mice (**E**) using ELISA, following the same treatment protocols as above. (**F**) The MLMVECs’ permeability was assessed after treatment with PM_2.5_ (1 mg/mL) in the presence or absence of the p38 MAPK inhibitor SB203580 (10 μM). (**G**) Cell viability was determined through the MTT assay in the MLMVECs treated with increasing concentrations of 3-DSC (up to 50 μM). Data are presented as the mean ± SD from at least three independent experiments. * *p* < 0.05 compared to the PM_2.5_-treated group. CNTL, control group.

PM_2.5_ is known to compromise the vascular barrier’s integrity [22,23,24], prompting the use of a vascular permeability assay to investigate the protective effects of 3-DSC on the MLMVECs (Figure 1). After exposure to PM_2.5_ (1 mg/mL for 6 h), the cells were treated with 3-DSC for an additional 6 h, resulting in a dose-dependent reduction in PM_2.5_-induced hyperpermeability (Figure 1A). These findings were validated further through in vivo experiments, where 3-DSC significantly reduced peritoneal dye leakage (Figure 1B). The wet-to-dry ratio (W/D) for lung weight served as an indicator for evaluating the impact of 3-DSC on PM_2.5_-induced lung injury. This ratio increased in the PM_2.5_-exposed group (Figure 1C) but decreased with the 3-DSC treatment. Given that inflammatory-protein-induced vascular disruption is regulated by p38 MAPK signaling pathways [25,26], this study next examined the impact of PM_2.5_ and 3-DSC on p38 MAPK activation. Treatment with PM_2.5_ increased phosphorylated p38 levels, which were substantially suppressed by 3-DSC in both the MLMVECs (Figure 1D) and the mice (Figure 1E). Additionally, PM_2.5_-induced endothelial hyperpermeability was shown to be mediated by p38 MAPK activation in the MLMVECs (Figure 1F). Toxicity testing revealed that 3-DSC had no adverse effects on cell viability at concentrations up to 50 µM in the purified MLMVECs (Figure 1G).

### 3.2. The Effects of 3-DSC on PM_2.5_-Stimulated ROS Generation in the MLMVECs

**Figure 2:**
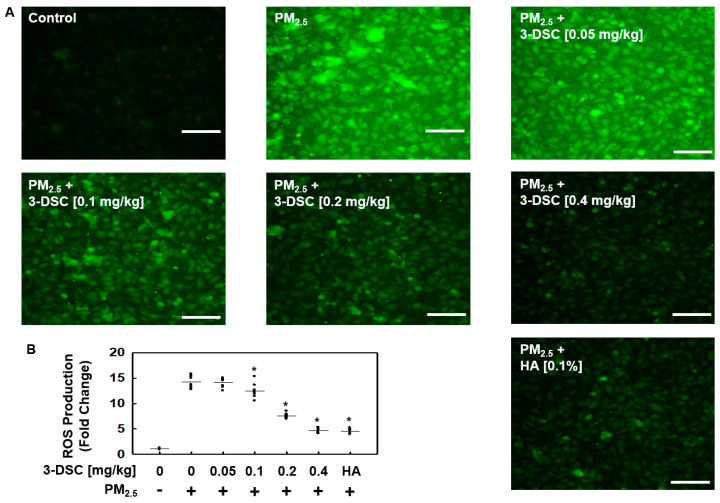
The effects of 3-DSC on the PM-induced generation of reactive oxygen species (ROS). Following a 10-day oral regimen of 3-DSC or hyaluronan (HA; 0.1%), the mice received intratracheal PM_2.5_ (1 mg/kg in 100 µL of saline) over the same period. (**A**) MLMVECs were isolated from mice orally treated with 3-DSC (0.05, 0.1, 0.2, or 0.4 mg/kg) or hyaluronan (HA; 0.1%) daily for 10 days, followed by the intratracheal instillation of PM_2.5_ (1 mg/kg in 100 μL of saline) for 10 days. After treatment, the cells (>90% confluence in 35 mm dishes) were incubated with 10 μM 2′,7′-dichlorofluorescein diacetate (DCFDA) for 30 min to detect intracellular ROS. (**B**) ROS levels were visualized and quantified through fluorescence microscopy. Representative images from each experimental group are shown (n = 5 mice per group). The data are presented as the mean ± SD from at least three independent experiments. * *p* < 0.05 compared to the PM_2.5_-exposed group. Scale bar: 100 μm.

Earlier research has demonstrated that PM exposure leads to significant intracellular oxidative stress due to mitochondrial dysfunction [13,23,27,28]. In the present study, 3-DSC effectively suppressed the generation of the ROS induced by PM_2.5_, as confirmed by the DCFDA oxidation assay (Figure 2). By acting as a ROS scavenger in the endothelial cells, 3-DSC prevented the buildup of PM-induced ROS and substantially mitigated the endothelial barrier disruption caused by PM_2.5_ exposure.

### 3.3. The Effects of 3-DSC on Akt Phosphorylation and Endothelial Cell Barrier Function

**Figure 3:**
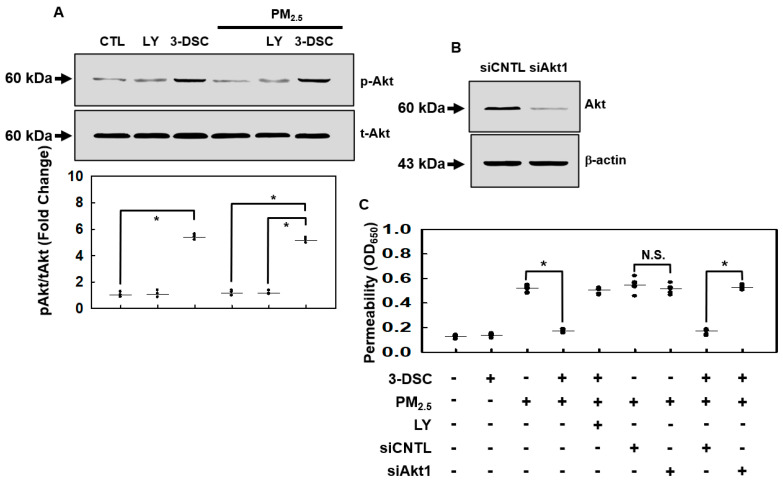
The effects of 3-DSC on Akt activation and endothelial cell barrier function under PM_2.5_ exposure. (**A**) MLMVECs were pretreated with 3-DSC (20 μM) for 6 h, followed by exposure to PM_2.5_ (1 mg/mL) for an additional 6 h. The phosphorylated Akt (p-Akt) and total Akt levels were analyzed through Western blotting, with representative blots and quantitative data (mean ± SD, n = 3 independent experiments) shown. (**B**) MLMVECs were transfected with Akt1 siRNA (100 nM) or control siRNA (100 nM) for 48 h. Akt protein expression was determined through Western blotting, using β-actin as a loading control. (**C**) The MLMVECs were pretreated with the PI3K inhibitor LY294002 (10 μM, 1 h), Akt1 siRNA, or control siRNA and then treated with 3-DSC (20 μM, 6 h) and exposed to PM_2.5_ (1 mg/mL, 6 h). Endothelial permeability was assessed using the Evans-blue-bound albumin transwell assay. The data are presented as the mean ± SD from at least three independent experiments. * *p* < 0.05; N.S., not significant.

The phosphatidylinositol 3-phosphate (PI3K)/Akt pathway plays a central role in regulating cell survival, proliferation, and metabolic processes in mammalian systems [29,30]. To assess whether 3-DSC modulates Akt signaling under PM_2.5_ exposure, we analyzed its effects on Akt phosphorylation. In the MLMVECs, 3-DSC robustly activated Akt phosphorylation, an effect reversed by the PI3K inhibitor LY294002 (Figure 3A). Notably, 3-DSC maintained Akt activation even after the PM_2.5_ treatment (1 mg/mL, 6 h). The protective effect of 3-DSC against PM_2.5_-induced endothelial barrier disruption was largely abolished by LY294002 (Figure 3C). To confirm Akt’s role, Akt1, the predominant endothelial Akt isoform [31], was silenced using siRNA, which reduced Akt’s protein levels (Figure 3B) and eliminated 3-DSC’s ability to counteract PM_2.5_-induced barrier dysfunction (Figure 3C). These findings demonstrate that 3-DSC mitigates PM_2.5_-mediated vascular damage in MLMVECs by enhancing Akt phosphorylation.

### 3.4. The Effects of 3-DSC on PM_2.5_-Induced Pulmonary Inflammation

**Figure 4:**
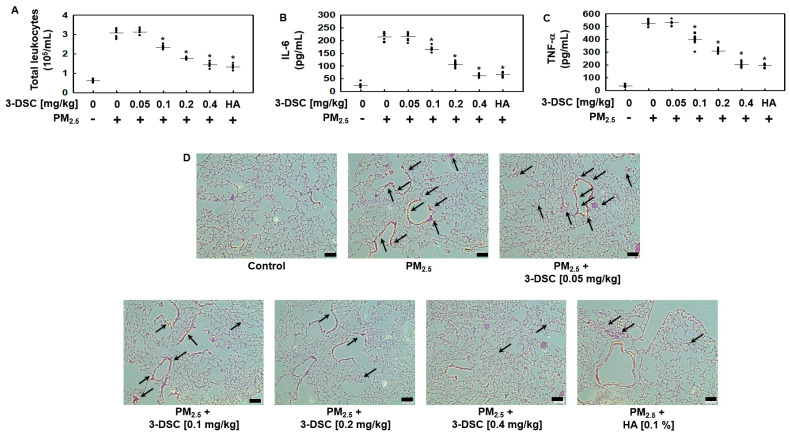
The effects of 3-DSC on particulate-matter-induced pulmonary inflammation and injury. (**A**–**C**) Male Balb/c mice (7 weeks old, n = 5 per group) were orally administered 3-DSC (0.05, 0.1, 0.2, or 0.4 mg/kg) or HA (0.1%) daily for 10 days. The mice were then intratracheally exposed to PM_2.5_ (1 mg/kg in 100 μL saline) for 10 days. Bronchoalveolar lavage (BAL) fluid was collected to assess (**A**) total leukocyte counts, (**B**) interleukin-6 (IL-6) levels, and (**C**) tumor necrosis factor-alpha (TNF-α) levels using ELISA. (**D**) Lung tissue sections were stained with hematoxylin and eosin (H&E) to evaluate the histopathological changes and leukocyte infiltration (arrows). Representative images from each group are shown (scale bar: 160 μm). The data are presented as the mean ± SD from at least three independent experiments. * *p* < 0.05 compared to the PM_2.5_-exposed group.

Given the observed ability of 3-DSC to mitigate PM_2.5_-induced vascular barrier dysfunction in vivo (Figure 2), we assessed its therapeutic potential against PM_2.5_-triggered pulmonary inflammation and tissue damage further. Exposure to PM_2.5_ elevated the inflammatory leukocyte counts in the bronchoalveolar lavage (BAL) fluid, a response effectively counteracted by 3-DSC administration (Figure 4A). The compound also suppressed PM_2.5_-induced secretion of the proinflammatory cytokines IL-6 (Figure 4B) and TNF-α (Figure 4C) in the BAL fluid. Histological evaluation of the lung tissues revealed that 3-DSC significantly reduced PM_2.5_-driven leukocyte infiltration (Figure 4D). Together, these results demonstrate that 3-DSC protects against PM_2.5_-induced lung injury by stabilizing vascular integrity, curbing immune cell recruitment, and dampening cytokine-mediated inflammatory cascades.

## 4. Discussion

Vascular inflammatory diseases, such as sepsis, pulmonary disorders, atherosclerosis, and severe inflammatory syndromes, are often characterized by endothelial barrier dysfunction and heightened vascular permeability [32,33]. Protecting the endothelial barrier offers significant therapeutic potential. Airborne particulate matter, rich in transition metals and polycyclic aromatic hydrocarbons, triggers excessive ROS production in the lung tissues, particularly within the vascular endothelium, which is highly vulnerable to oxidative stress and plays a pivotal role in the progression of vascular pathologies [34,35]. The endothelium serves as a selective barrier regulating plasma and cellular passage into the surrounding tissues, but inflammatory mediators can transiently or irreversibly disrupt this function, leading to increased permeability, fluid leakage, and tissue damage [36,37]. Strategies to prevent such disruptions could improve the outcomes for patients with inflammatory conditions. This study demonstrated that 3-DSC effectively counteracted PM_2.5_-induced endothelial barrier dysfunction and reduced lung vascular leakage by modulating the p38 MAPK signaling pathway, highlighting its potential role in preserving endothelial integrity under particulate matter exposure.

Extensive epidemiological research highlights the harmful effects of ambient particulate matter (PM) on cardiopulmonary health, linking PM exposure to increased morbidity and mortality from cardiovascular and respiratory diseases [38,39]. Proposed mechanisms for these adverse effects include oxidative stress and inflammation at both the pulmonary and systemic levels, the activation of coagulation pathways, and disruptions in cardiac autonomic function [40,41,42]. Recent studies have shown that PM exposure provokes significant lung inflammation and vascular hyperpermeability, characterized by endothelial barrier dysfunction, protein leakage, neutrophil infiltration, and elevated proinflammatory cytokine levels [35,43,44]. These changes contribute to systemic inflammation, exacerbating conditions like asthma, chronic obstructive pulmonary disease (COPD), cardiac arrhythmia, and heart failure. In this study, 3-DSC effectively scavenged PM_2.5_-induced ROS; inhibited oxidative-stress-mediated activation of p38 MAPK and Akt; and reduced vascular leakage, leukocyte infiltration, and proinflammatory cytokine release in the bronchoalveolar lavage fluid of PM-exposed mice. These findings suggest that 3-DSC may serve as a protective agent against ROS-driven pulmonary inflammation caused by air pollution.

Hydroxyl radicals, highly reactive byproducts of oxidative processes, induce molecular damage and drive chain reactions that disrupt cellular components [45]. When the ROS production overwhelms antioxidant defenses, the redox imbalance triggers inflammatory cascades [46,47] and contributes to respiratory inflammation and airway hypersensitivity [48]. This study highlights 3-DSC’s ability to counteract PM-induced ROS generation in the respiratory system. Ultrafine PM particles (≤2.5 µm) translocate from the alveoli to the pulmonary vasculature, exacerbating systemic vascular dysfunction [49,50], while infiltrating neutrophils amplify ROS production, perpetuating inflammatory cycles [51]. Here, the ROS generated by PM exposure triggered p38 MAPK activation in the lung endothelial cells, leading to cytoskeletal rearrangements, marked by stress fiber formation and paracellular gaps, that compromised barrier integrity [23]. 3-DSC mitigated PM-induced vascular leakage by suppressing p38 MAPK activation, functioning as an ROS scavenger to preserve endothelial stability under oxidative stress, underscoring its therapeutic potential against particulate-driven lung injury.

The present study provides compelling evidence that 3-DSC, a natural chalcone derivative, offers significant protection against PM_2.5_-induced pulmonary injury by preserving endothelial barrier function, suppressing oxidative stress, and attenuating inflammation. Our results demonstrate that 3-DSC effectively reduces PM_2.5_-induced vascular hyperpermeability both in vitro and in vivo, as evidenced by the decreased leakage of Evans blue dye and improved endothelial integrity. These findings are consistent with prior studies highlighting the central role of oxidative stress and inflammation in PM_2.5_-mediated lung injury and further support the therapeutic potential of natural antioxidants to mitigate environmental-pollutant-induced damage. Mechanistically, 3-DSC was shown to neutralize the ROS generated by PM_2.5_ exposure, thereby inhibiting downstream activation of the p38 MAPK pathway—a key driver of endothelial dysfunction and inflammatory responses. Additionally, 3-DSC enhanced Akt signaling, which is crucial to maintaining vascular barrier stability. The dual modulation of both the p38 MAPK and Akt pathways by 3-DSC underscores its multifaceted protective effects and distinguishes it from other agents that target only a single pathway. Importantly, the observed reduction in proinflammatory cytokines (IL-6 and TNF-α) and leukocyte infiltration in the lungs further substantiates the anti-inflammatory efficacy of 3-DSC. These findings have important implications for public health, particularly in regions with high levels of air pollution. By demonstrating the efficacy of 3-DSC in both cellular and animal models, this study lays the groundwork for future translational research and potential clinical application. Moreover, the use of hyaluronan as a positive control validates the experimental approach and reinforces the robustness of the observed effects. Collectively, our data suggest that 3-DSC may serve as a promising candidate for the prevention or treatment of PM_2.5_-induced pulmonary disorders, warranting further investigation in clinical settings. Therefore, this study not only elucidates the molecular mechanisms underlying the protective effects of 3-DSC but also expands the repertoire of potential interventions against air-pollution-induced lung injury. Future studies should explore the long-term safety, pharmacokinetics, and efficacy of 3-DSC in diverse populations and under chronic exposure conditions to fully realize its therapeutic potential.

Despite its promising results, several limitations of this study should be acknowledged to provide a balanced perspective and guide future research. First, the experiments were conducted using a relatively small sample size, particularly in the animal studies, which may limit the generalizability of the findings. Second, while the in vitro and in vivo models employed are well-established for studying PM_2.5_-induced lung injury, they may not fully recapitulate the complexity of human exposure scenarios, including chronic and low-dose exposure over extended periods. Additionally, this study primarily focused on acute responses to PM_2.5_ and did not assess the long-term effects or potential toxicity of repeated 3-DSC administration. The use of a single animal species and sex (male Balb/c mice) further restricts the applicability of the results to broader populations. Moreover, although key signaling pathways such as p38 MAPK and Akt were investigated, other relevant molecular mechanisms and potential off-target effects of 3-DSC remain to be explored. Finally, while this study demonstrated the efficacy of 3-DSC against PM_2.5_-induced injury, it did not compare its effects with other known antioxidants or anti-inflammatory agents, which could provide additional context for its relative therapeutic value. Addressing these limitations in future studies, including larger and more diverse cohorts, chronic exposure models, and comprehensive mechanistic analyses, will be essential to validate and extend the current findings.

## 5. Conclusions

Our research demonstrated that 3-DSC effectively protected against PM-induced endothelial barrier dysfunction and vascular hyperpermeability by simultaneously scavenging ROS and activating the Akt signaling pathway. These dual actions suggest that 3-DSC may help alleviate pulmonary inflammation driven by the ROS overproduction caused by PM air pollution.

## Data Availability

The data presented in this study are available upon reasonable request from the corresponding author.

## Abbreviations

The following abbreviations are used in this manuscript:

PM	particulate matter
3-DSC	3-deoxysappanchalcone
ROS	reactive oxygen species
BAL	bronchoalveolar lavage fluid
MLMVECs	mouse lung microvascular endothelial cells
IL	interleukin
MTT	3-(4,5-dimethylthiazol-2-yl)-2,5-diphenyltetrazolium bromide
DMSO	dimethyl sulfoxide

## References

[1] Wang Y., Wang Z., Jiang J., Guo T., Chen S., Li Z., Yuan Z., Lin Q., Du Z., Wei J. (2024). The effect of long-term particulate matter exposure on respiratory mortality: Cohort study in china. JMIR Public Health Surveill..

[2] Pryor J.T., Cowley L.O., Simonds S.E. (2022). The physiological effects of air pollution: Particulate matter, physiology and disease. Front. Public Health.

[3] Viher Hrzenjak V., Kukec A., Erzen I., Stanimirovic D. (2020). Effects of ultrafine particles in ambient air on primary health care consultations for diabetes in children and elderly population in ljubljana, slovenia: A 5-year time-trend study. Int. J. Environ. Res. Public Health.

[4] Schraufnagel D.E. (2020). The health effects of ultrafine particles. Exp. Mol. Med..

[5] Abdel-Aziz S., Aeron A., Kahil T. (2016). Health Benefits and Possible Risks of Herbal Medicine.

[6] Fu L.C., Huang X.A., Lai Z.Y., Hu Y.J., Liu H.J., Cai X.L. (2008). A new 3-benzylchroman derivative from sappan lignum (*Caesalpinia sappan*). Molecules.

[7] Badami S., Moorkoth S., Rai S.R., Kannan E., Bhojraj S. (2003). Antioxidant activity of *Caesalpinia sappan* heartwood. Biol. Pharm. Bull..

[8] Jung E.G., Han K.I., Kwon H.J., Patnaik B.B., Kim W.J., Hur G.M., Nam K.W., Han M.D. (2015). Anti-inflammatory activity of sappanchalcone isolated from *Caesalpinia sappan* L. In a collagen-induced arthritis mouse model. Arch. Pharm. Res..

[9] Kim C., Kim B. (2018). Anti-cancer natural products and their bioactive compounds inducing er stress-mediated apoptosis: A review. Nutrients.

[10] Liu A.L., Shu S.H., Qin H.L., Lee S.M., Wang Y.T., Du G.H. (2009). In vitro anti-influenza viral activities of constituents from *Caesalpinia sappan*. Planta Med..

[11] Yodsaoue O., Cheenpracha S., Karalai C., Ponglimanont C., Tewtrakul S. (2009). Anti-allergic activity of principles from the roots and heartwood of *Caesalpinia sappan* on antigen-induced beta-hexosaminidase release. Phytother. Res..

[12] Sireeratawong S., Piyabhan P., Singhalak T., Wongkrajang Y., Temsiririrkkul R., Punsrirat J., Ruangwises N., Saraya S., Lerdvuthisopon N., Jaijoy K. (2010). Toxicity evaluation of sappan wood extract in rats. J. Med. Assoc. Thail..

[13] Xu C., Shi Q., Zhang L., Zhao H. (2018). High molecular weight hyaluronan attenuates fine particulate matter-induced acute lung injury through inhibition of ros-ask1-p38/jnk-mediated epithelial apoptosis. Environ. Toxicol. Pharmacol..

[14] Wang H., Song L., Ju W., Wang X., Dong L., Zhang Y., Ya P., Yang C., Li F. (2017). The acute airway inflammation induced by PM_2.5_ exposure and the treatment of essential oils in balb/c mice. Sci. Rep..

[15] Kovacs-Kasa A., Varn M.N., Verin A.D., Gonzales J.N. (2017). Method for the culture of mouse pulmonary microvascular endothelial cells. Sci. Pages Pulmonol..

[16] Cho S., Park Y.J., Lee J., Bae J.-S. (2024). Suppressive activities of lupeol on sepsis mouse model. Biotechnol. Bioprocess. Eng..

[17] Baek D.H., Kim G.O., Choi H.J., Yun M.Y., Park D.H., Song G.Y., Bae J.S. (2023). Inhibitory activities of gdx-365 on hmgb1-mediated septic responses. Biotechnol. Bioprocess Eng..

[18] Zhang L., Wang M.C. (2018). Growth inhibitory effect of mangiferin on thyroid cancer cell line tpc1. Biotechnol. Bioprocess Eng..

[19] Jang M.H., Kang N.H., Mukherjee S., Yun J.W. (2018). Theobromine, a methylxanthine in cocoa bean, stimulates thermogenesis by inducing white fat browning and activating brown adipocytes. Biotechnol. Bioprocess Eng..

[20] Piao M.J., Ahn M.J., Kang K.A., Ryu Y.S., Hyun Y.J., Shilnikova K., Zhen A.X., Jeong J.W., Choi Y.H., Kang H.K. (2018). Particulate matter 2.5 damages skin cells by inducing oxidative stress, subcellular organelle dysfunction, and apoptosis. Arch. Toxicol..

[21] Ozdulger A., Cinel I., Koksel O., Cinel L., Avlan D., Unlu A., Okcu H., Dikmengil M., Oral U. (2003). The protective effect of n-acetylcysteine on apoptotic lung injury in cecal ligation and puncture-induced sepsis model. Shock.

[22] Wang T., Shimizu Y., Wu X., Kelly G.T., Xu X., Wang L., Qian Z., Chen Y., Garcia J.G.N. (2017). Particulate matter disrupts human lung endothelial cell barrier integrity via rho-dependent pathways. Pulm. Circ..

[23] Wang T., Chiang E.T., Moreno-Vinasco L., Lang G.D., Pendyala S., Samet J.M., Geyh A.S., Breysse P.N., Chillrud S.N., Natarajan V. (2010). Particulate matter disrupts human lung endothelial barrier integrity via ros- and p38 mapk-dependent pathways. Am. J. Respir. Cell Mol. Biol..

[24] Long Y.M., Yang X.Z., Yang Q.Q., Clermont A.C., Yin Y.G., Liu G.L., Hu L.G., Liu Q., Zhou Q.F., Liu Q.S. (2020). PM_2.5_ induces vascular permeability increase through activating mapk/erk signaling pathway and ros generation. J. Hazard. Mater..

[25] Wang T., Liu C., Pan L.H., Liu Z., Li C.L., Lin J.Y., He Y., Xiao J.Y., Wu S., Qin Y. (2020). Inhibition of p38 mapk mitigates lung ischemia reperfusion injury by reducing blood-air barrier hyperpermeability. Front. Pharmacol..

[26] Li L., Hu J., He T., Zhang Q., Yang X., Lan X., Zhang D., Mei H., Chen B., Huang Y. (2015). P38/mapk contributes to endothelial barrier dysfunction via map4 phosphorylation-dependent microtubule disassembly in inflammation-induced acute lung injury. Sci. Rep..

[27] Zhao Y., Usatyuk P.V., Gorshkova I.A., He D., Wang T., Moreno-Vinasco L., Geyh A.S., Breysse P.N., Samet J.M., Spannhake E.W. (2009). Regulation of cox-2 expression and il-6 release by particulate matter in airway epithelial cells. Am. J. Respir. Cell Mol. Biol..

[28] Gualtieri M., Longhin E., Mattioli M., Mantecca P., Tinaglia V., Mangano E., Proverbio M.C., Bestetti G., Camatini M., Battaglia C. (2012). Gene expression profiling of a549 cells exposed to milan PM_2.5_. Toxicol. Lett..

[29] Han B., Lin X., Hu H. (2024). Regulation of pi3k signaling in cancer metabolism and pi3k-targeting therapy. Transl. Breast Cancer Res..

[30] He Y., Sun M.M., Zhang G.G., Yang J., Chen K.S., Xu W.W., Li B. (2021). Targeting pi3k/akt signal transduction for cancer therapy. Signal Transduct. Target. Ther..

[31] Shiojima I., Walsh K. (2002). Role of akt signaling in vascular homeostasis and angiogenesis. Circ. Res..

[32] Prasad M., Leon M., Lerman L.O., Lerman A. (2021). Viral endothelial dysfunction: A unifying mechanism for COVID-19. Mayo Clin. Proc..

[33] Qiao X., Yin J., Zheng Z., Li L., Feng X. (2024). Endothelial cell dynamics in sepsis-induced acute lung injury and acute respiratory distress syndrome: Pathogenesis and therapeutic implications. Cell Commun. Signal.

[34] Minjares M., Wu W., Wang J.M. (2023). Oxidative stress and micrornas in endothelial cells under metabolic disorders. Cells.

[35] Almeida-Silva M., Cardoso J., Alemão C., Santos S., Monteiro A., Manteigas V., Marques-Ramos A. (2022). Impact of particles on pulmonary endothelial cells. Toxics.

[36] Su Y., Lucas R., Fulton D.J.R., Verin A.D. (2024). Mechanisms of pulmonary endothelial barrier dysfunction in acute lung injury and acute respiratory distress syndrome. Chin. Med. J. Pulm. Crit. Care Med..

[37] Jin Y., Ji W., Yang H., Chen S., Zhang W., Duan G. (2020). Endothelial activation and dysfunction in COVID-19: From basic mechanisms to potential therapeutic approaches. Signal Transduct. Target. Ther..

[38] Liu C., Chan K.H., Lv J., Lam H., Newell K., Meng X., Liu Y., Chen R., Kartsonaki C., Wright N. (2022). Long-term exposure to ambient fine particulate matter and incidence of major cardiovascular diseases: A prospective study of 0.5 million adults in china. Environ. Sci. Technol..

[39] Guo J., Chai G., Song X., Hui X., Li Z., Feng X., Yang K. (2023). Long-term exposure to particulate matter on cardiovascular and respiratory diseases in low- and middle-income countries: A systematic review and meta-analysis. Front. Public Health.

[40] Schicker B., Kuhn M., Fehr R., Asmis L.M., Karagiannidis C., Reinhart W.H. (2009). Particulate matter inhalation during hay storing activity induces systemic inflammation and platelet aggregation. Eur. J. Appl. Physiol..

[41] Mutlu G.M., Green D., Bellmeyer A., Baker C.M., Burgess Z., Rajamannan N., Christman J.W., Foiles N., Kamp D.W., Ghio A.J. (2007). Ambient particulate matter accelerates coagulation via an il-6-dependent pathway. J. Clin. Investig..

[42] Baccarelli A., Cassano P.A., Litonjua A., Park S.K., Suh H., Sparrow D., Vokonas P., Schwartz J. (2008). Cardiac autonomic dysfunction: Effects from particulate air pollution and protection by dietary methyl nutrients and metabolic polymorphisms. Circulation.

[43] Long M.H., Zhu X.M., Wang Q., Chen Y., Gan X.D., Li F., Fu W.L., Xing W.W., Xu D.Q., Xu D.G. (2020). PM_2.5_ exposure induces vascular dysfunction via no generated by inos in lung of apoe-/- mouse. Int. J. Biol. Sci..

[44] Valderrama A., Ortiz-Hernández P., Agraz-Cibrián J.M., Tabares-Guevara J.H., Gómez D.M., Zambrano-Zaragoza J.F., Taborda N.A., Hernandez J.C. (2022). Particulate matter (PM_10_) induces in vitro activation of human neutrophils, and lung histopathological alterations in a mouse model. Sci. Rep..

[45] Sun Y., Yin Y., Zhang J., Yu H., Wang X., Wu J., Xue Y. (2008). Hydroxyl radical generation and oxidative stress in carassius auratus liver, exposed to pyrene. Ecotoxicol. Environ. Saf..

[46] Garcon G., Dagher Z., Zerimech F., Ledoux F., Courcot D., Aboukais A., Puskaric E., Shirali P. (2006). Dunkerque city air pollution particulate matter-induced cytotoxicity, oxidative stress and inflammation in human epithelial lung cells (l132) in culture. Toxicol. Vitr..

[47] Pozzi R., De Berardis B., Paoletti L., Guastadisegni C. (2003). Inflammatory mediators induced by coarse (PM_2.5–10_) and fine (PM_2.5_) urban air particles in raw 264.7 cells. Toxicology.

[48] Hulsmann A.R., Raatgeep H.R., den Hollander J.C., Stijnen T., Saxena P.R., Kerrebijn K.F., de Jongste J.C. (1994). Oxidative epithelial damage produces hyperresponsiveness of human peripheral airways. Am. J. Respir. Crit. Care Med..

[49] Kreyling W.G., Semmler M., Erbe F., Mayer P., Takenaka S., Schulz H., Oberdorster G., Ziesenis A. (2002). Translocation of ultrafine insoluble iridium particles from lung epithelium to extrapulmonary organs is size dependent but very low. J. Toxicol. Environ. Health A.

[50] Karoly E.D., Li Z., Dailey L.A., Hyseni X., Huang Y.C. (2007). Up-regulation of tissue factor in human pulmonary artery endothelial cells after ultrafine particle exposure. Environ. Health Perspect..

[51] Walters D.M., Breysse P.N., Wills-Karp M. (2001). Ambient urban baltimore particulate-induced airway hyperresponsiveness and inflammation in mice. Am. J. Respir. Crit. Care Med..

